# Supplementary material for: HIV Promotes Atherosclerosis via Circulating Extracellular Vesicle MicroRNAs
Source: Int J Mol Sci. 2023 Apr 20;24(8):7567. doi: 10.3390/ijms24087567 (PMC10146407; doi:10.3390/ijms24087567)
Supplement: Supplementary file 1 [file ijms-24-07567-s001.zip › ijms-2288861-supplementary.pdf]

SUPPLEMENTAL MATERIAL

HIV Promotes Atherosclerosis via Circulating Extracellular Vesicle MicroRNAs

Andrea Da Fonseca Ferreira [1], Jianqin Wei [1,2], Lukun Zhang [1], Conrad J. Macon [2], Bernard Degnan [1], Dushyantha Jayaweera [2], Joshua M. Hare [1,2], Michael A. Kolber [2], Michael Bellio [1], Aisha Khan [1], Yue Pan [3], Derek M. Dykxhoorn [4,5], Liyong Wang [4,5,*] and Chunming Dong [1,2,6,*]

[1] Interdisciplinary Stem Cell Institute, University of Miami Miller School of Medicine, Miami, FL 33136, USA
[2] Department of Medicine, University of Miami Miller School of Medicine, Miami, FL 33136, USA
[3] Biostatistics Division, Department of Public Health Sciences, University of Miami Miller School of Medicine, Miami, FL 33136, USA
[4] John T. Macdonald Foundation Department of Human Genetics, University of Miami Miller School of Medicine, Miami, FL 33136, USA
[5] John P. Hussman Institute for Human Genomics, University of Miami Miller School of Medicine, Miami, FL 33136, USA
[6] Section of Cardiology, Department of Medicine, Miami VA Health System, University of Miami, Miami, FL 33146, USA
* Correspondence: lwang1@med.miami.edu (L.W.); cdong3@med.miami.edu (C.D.); Tel.: +1-305-243-2377 (L.W.); +1-305-243-4706 (C.D.)

SUMMARY

Supplemental Figures…….……………………………………………………………...3

Supplemental Tables…………………………………………………………………….9

Full Western blot membranes….……………………………………………………….13

Supplemental References……………………………………………………………….14

SUPPLEMENTAL FIGURES

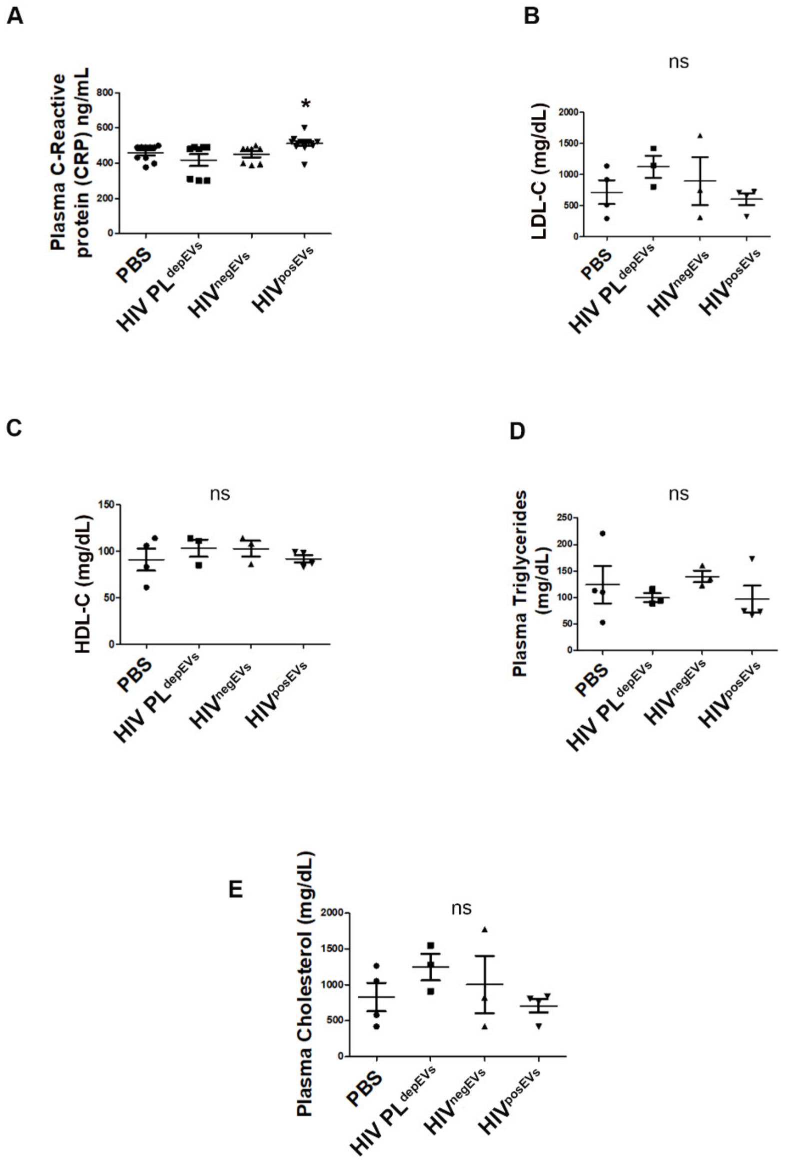

Supplemental Figure S1. Elevated C reactive protein level in mice treated with HIV^{posEVs} and similar lipoprotein profiles in mice with different treatments. **A.** C Reactive protein level in the serum was higher in mice treated with HIV^{posEVs} compared to animals treated $HIVPL^{depEVs}$, HIV^{negEVs} and PBS. **B, C, D, E.** Serum lipoprotein panel assays show no difference between different treatment groups (*p<0.05).

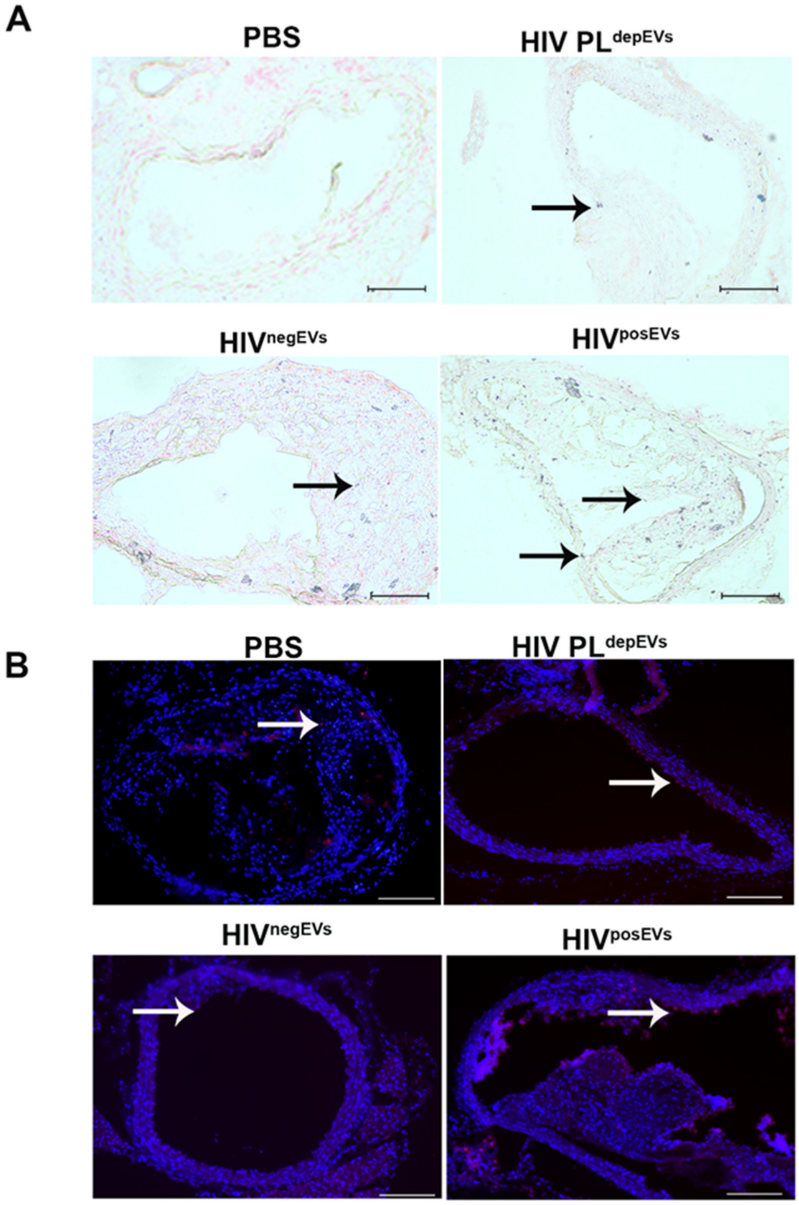

Supplemental Figure S2. Enlargement of representative images from Figure 3. HIVposEVs treatment results in impaired vascular repair/rejuvenation in vivo. (A). Aorta sections submitted to β-gal staining. Positive cells are stained in blue. (B). Aorta sections submitted to TUNEL staining. Positive cells are stained in red. Arrows show examples of positive cells for each staining type. Scale bars: 200 μm.

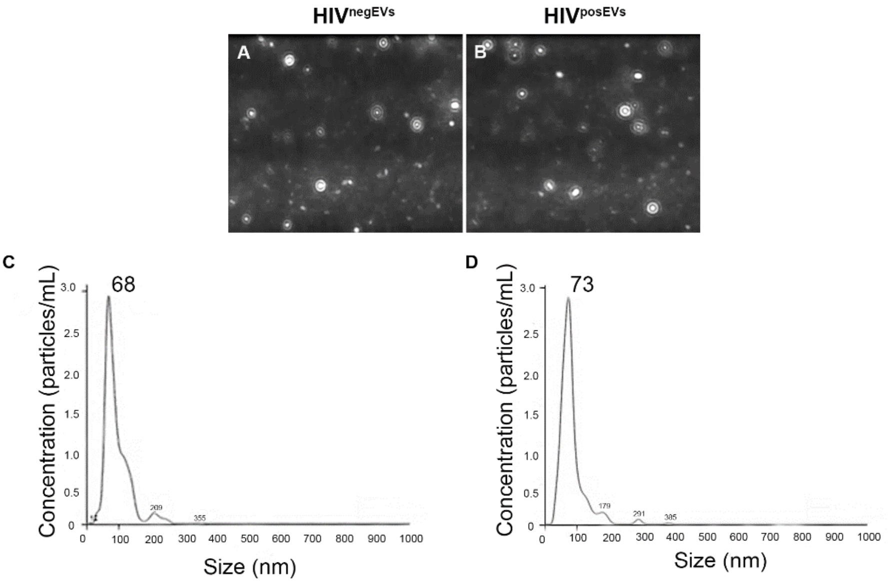

E

Sample	Average Size (nm)	Protein concentration (mg/mL)	Particle Concentration (Particle/mL)	Ratio particle:protein	Ratio particle:RNA	Ratio particle:lipid
HIV^{negEVs}	83±41.1	1.42±0.4	1.75E+10	1.23E+10	4.39E+10	1.12E+10
HIV^{posEVs}	92.1±41.6	1.47±0.5	1.81E+10	1.24E+10	5.34E+10	1.13E+10
p-value	0.38	1.00	0.82	0.84	0.79	0.44

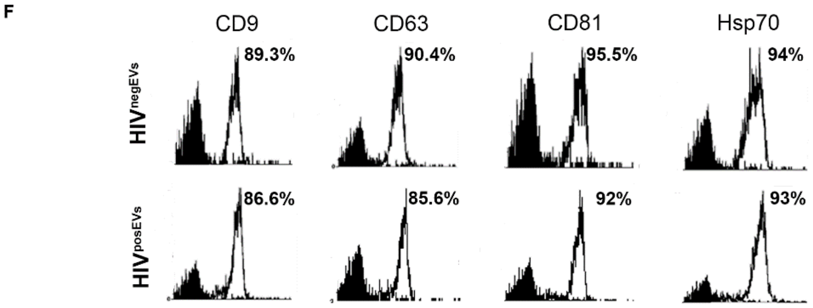

G

Sample	CD9 (%)	CD63 (%)	CD81 (%)	*Hsp70 (%)*
HIV^{negEVs}	89.2±6.0	90.3±4.6	95.4±3.4	93.1±3.9
HIV^{posEVs}	86.5±4.5	85.6±7.3	91.8±1.6	93.9±2.2
p-value	0.43	0.42	0.22	0.54

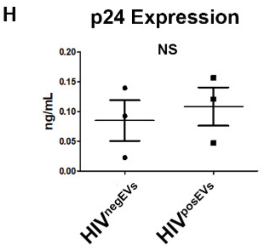

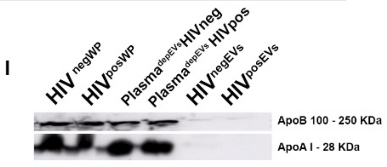

Supplemental Figure S3. NTA and flow cytometry analyses demonstrate similar EV populations in PLHIV and HIV negative individuals. **A&B.** Representative images of NTA of HIV^{posEVs} and HIV^{negEVs}, respectively. **C&D.** Particle diameter distribution histograms show similar sizes of EVs present in samples of the two groups, and the majorities of the particles concentrate in sole peaks of diameter lengths. **E**. Average diameter size, total protein concentration for HIV^{posEVs} and HIV^{negEVs} show no differences. Ratios are also shown. Ratios are calculated by dividing the number of particles of a sample by its concentration of protein, RNA or total lipids assessed by standard protocols as suggested by the International Society for Extracellular Vesicles (ISEV). **F**. Representative flow cytometry histograms show that the majority of EVs from both groups similarly express surface markers typical for EVs, including CD63, CD81, CD9 and Hsp70. **G.** Mean ± SD for expression of EV markers measured by Flow Cytometry. **H.** p24 expression assessed by ELISA shows no difference between the two groups. **I.** ApoB 100 and ApoA1 Western blots of whole plasma ($HIVneg^{WP}$ and HIV pos^{WP}), $plasma^{depEVs}$ from both groups and EVs preparations ($HIVneg^{EVs}$ and HIV pos^{EVs}) show no contamination of lipoproteins in EV preparations. All experiments were performed in triplicates.

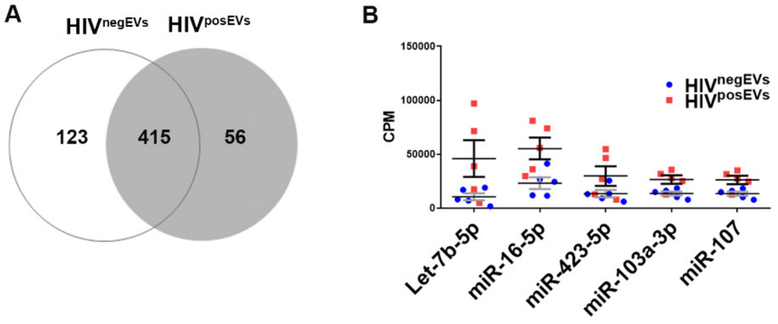

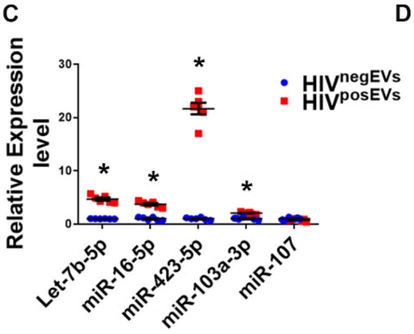

D

Disease/Function	P-value range
Cardiac Enlargement	1.72E-01 – 5.89E-04
Cardiac Fibrosis	3.14E-02 – 3.14E-02
Cardiac Necrosis/Cell Death	2.14E-01 – 3.19E-02

Supplemental Figure S4. sRNA-seq analyses of HIV^{posEVs} and HIV^{negEVs}. **(A)** Venn Diagram showing the number of miRs shared by the two groups (415); among the 415 shared miRs, 126 were upregulated and 49 downregulated in HIV^{posEVs} (240 were nonsignificantly regulated)The CPM for miRs only expressed in HIV^{posEVs} or in HIV^{negEVs} are too low for further analysis/investigation. (**B)** Dot plot showing CPM of the top 5 upregulated miRs in HIV^{posEVs} versus HIV^{negEVs}. **(C)** Four of top 5 overexpressed miRs in HIV^{posEVs} were validated by qRT-PCR (N=5 for each group). **(D)** Pathway analyses conducted with the aid of the software IPA(QIAGEN) implicate the involvement of the 4 validated candidate EV-miRs in cardiac enlargement, cardiac fibrosis and cardiac necrosis/cell death (*p* value ranges express higher level functions that contained multiple lower level functions) (*$p<0.05$).

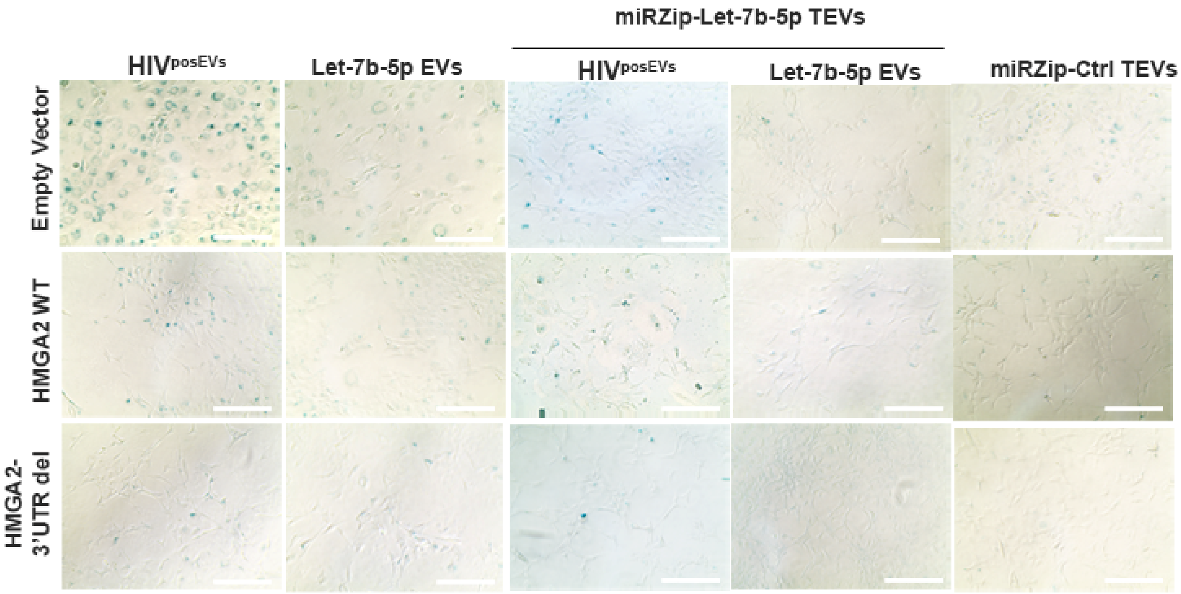

Supplemental Figure S5. Representative images from β-gal staining experiments detailed in Figure 7**.** HMGA2 with 3'UTR deletion attenuates HIV^{posEVs} effect in lin- BMC senescence. Scale bars: 200µm.

SUPPLEMENTAL TABLES

Supplemental Table S1. Demographics and clinical characteristics of study subjects.

Variable	HIV^{neg}	PLHIV	*P*
**Patients**	23	74	
**Age (year)**	40.7 ±12.1	47.8±10.1	*P* <0.05
**Male (%)**	11 (47.8%)	39 (52.7%)	
**cIMT (mm)**	0.72 ±0.12	0.98 ±0.36	*P* <0.05
**CFU**	5.94±2.27	2.33 ±1.23	*P* <0.001

Supplemental Table S1. Demographics and clinical characteristics of study subjects. Continuous variables expressed as mean ± SE; categorical variables are expressed as number and frequency (%). cIMT = Carotid Intima-Media Thickness Test; CFU = Colony Forming Units. $p<0.05$ was considered statistically significant for continuous variables.

Supplemental Table S2. **Validated candidate EV-miRs overrepresented in HIV^{posEVs} and their target genes.**

Rank	MicroRNA	Targets	Effect
#1	Let-7b-5p	ACVR1C,ADRB2,ADRB3 ARG2,BACH1,BCL2L1 CASP3,CCND1,CDKN1A CHRNA7,COL1A2,COL27A1 DCLRE1C,E2F6,EDN1,F2 FANCD2,FAS,FASLG,GLRX GNG5,HAND1,HMGA2,HMOX1,IGF1R IL10,IL13,ITGB3,KCNJ11,KRAS MAPK6,MDM4,MYC,MYCN,NRAS PDE12,POU2F1,PRDM1,PRIM1 PTGS2,RAS,RHOG,S100A8,SO1 TGFBR1,TGFBR3,THBS1,TLR4 TNFSF10,TP53, WNT1	High expression in congestive heart failure[1], myocardial infarction (MI)[2], atrial fibrillation[3], angiogenesis[4], and stroke[5].
#2	miR-16-5p	ACVR2B,AKT3,ALOX12 APLN,ARHGDIA,ATF6 BCL2,CACNA2D1,CCND1 CCNE1,CD28,CD40,CD80 CHEK1,DNAJB4,E2F3 E2F7,EGFR,F2,FGF2,FGF7 FGFR1,FLT3,GLRX,GNAI3 GRB2,HLA-DQB2,HMOX1 HSP90B1,HSPA1A/HSPA1B IGF1,IGF1R,IHH,IKBKB,IL15 INSR,ITGA2,JUN,KCNN4, LAMC1,MAP2K1,MAP2K4 MAPK3,MSH2,MYB,MYLK NPR3,PAFAH1B2,PANX1 PLD1,PLK1,PRIM1,PTGS2 RAF1,RPS6KA3,SGK1, SLC12A2,SLC2A3,SLC7A1 SMAD7,SMPD1,TLK1, TNFSF13B,VEGFA,VPS33B	High in PLHIV[6], Impacts EPCs function[7].
#3	miR-423-5p	ANGPT4,APOC2,ARHGDIA ARHGEF4,CACNA1B,CALM1 CASP9,CDKN1A,CHRND CRK,CSF1,CTSD,EIF4EBP1 GNAI2,HSPA1L,IL17A,KLK4 MAPK3,MEF2D,NGFR,NOX4 NPR3,PDCD1,PLA2G6,PLCB1 PML,PPARD,PRKACA,PTK2B PTPA,RAC2,RPL36,SERPIND1 SHC1,SLC9A1,SMARCC2 TP73,WNT9B	Involved in Coronary artery disease stability[8].

#4	miR-103-3p	AGO2,APLN,CACNA2D1<br>CCNE1,CD80,DNAJB4<br>FGFRL1,GRAP2,NPR3<br>P2RY12,PRKCE,PTEN<br>RCAN1,SLC23A1,SMPD1<br>TDG,TGFBR2	Promotes apoptosis[9], targets VEGF[10], promotes endothelial maladaptation[11], impairs tube formation[12].

Supplemental Table S2. sRNA-seq revealed top upregulated microRNAs in HIV^{posEVs} compared to HIV^{negEVs}. IPA target finder tool generated lists of putative targets for those microRNAs of interest. A literature search was conducted as to investigate effects of those microRNAs in lin^- BMCs and CVD.

Supplemental Table S3. Detection of expression of miR-Zip-Ctrl, miRZip-Let-7b-5p, and Let-7b-5p in TEV-treated lin$^-$ BMCs.

	miR-Zip-Ctrl		miR-Zip-Let-7b-5p		Let-7b-5p	
	U6	miR-Zip-Ctrl	U6	miR-Zip-Let-7b-5p	U6	Let-7b-5p
Empty Vector	20.62±0.35	35.48±0.38	22.3 ±0.36	35.02±1.61	19.71±0.038	36.01±0.97
HMGA2 WT	21.25±0.27	36.15±0.10	21.6±0.38	33.78±0.65	20.54±0.43	36.55±0.77
HMGA2-3' UTR del	23.41±0.42	38.09±0.22	22.8±0.60	34.80±0.31	20.71±0.93	37.36±0.64
Untreated Control	-	-	-	-	19.52±0.37	37.49±0.15

Supplemental Table S3. Lin$^-$ BMCs transduced with an empty control vector , WT HMGA2. or HMGA2-3'UTR del were incubated with miRZip-Let-7b-5p TEVs, miRZip-Ctrl TEVs or Let-7b-5p EVs. Un-transduced and untreated lin$^-$ BMCs were used as an additional control (Untreated Control). Since miRZip-Let-7b-5p and miRZip-Ctrl are synthetic oligos, no expression of those were expected in the untreated lin$^-$ BMC.

FULL WESTERN BLOT MEMBRANES

A. Figure 8

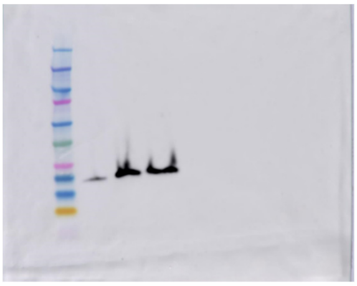

HMGA2

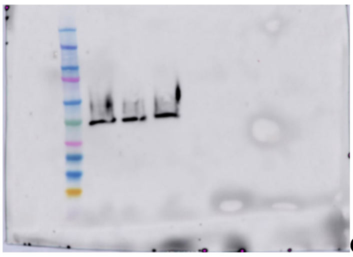

GAPDH

B. Supplemental Figure S2, I

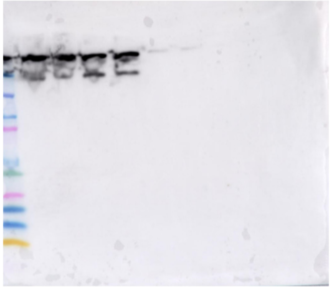

ApoB

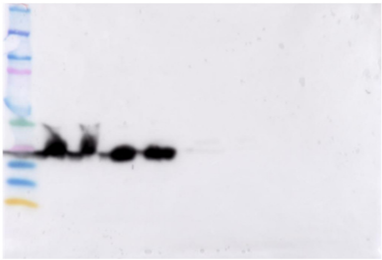

ApoA

Representative western blot membranes. Full western blot membranes used as representatives in this work. Membranes in **A** were used for Figure 8, and membranes in **B** were used for Supplemental Figure S2.

SUPPLEMENTAL REFERENCES

1. Bao MH, Feng X, Zhang YW, Lou XY, Cheng Y, Zhou HH. Let-7 in cardiovascular diseases, heart development and cardiovascular differentiation from stem cells. Int J Mol Sci. 2013;14(11):23086-23102. Published 2013 Nov 21. doi:10.3390/ijms141123086.
2. Long G, Wang F, Duan Q, et al. Circulating miR-30a, miR-195 and let-7b associated with acute myocardial infarction. PLoS One. 2012;7(12):e50926. doi:10.1371/journal.pone.0050926.
3. Mun D, Kim H, Kang JY, et al. Expression of miRNAs in circulating exosomes derived from patients with persistent atrial fibrillation. FASEB J. 2019;33(5):5979-5989. doi:10.1096/fj.201801758R.
4. Aday S, Hazan-Halevy I, Chamorro-Jorganes A, et al. Bioinspired artificial exosomes based on lipid nanoparticles carrying let-7b-5p promote angiogenesis in vitro and in vivo. Mol Ther. 2021;29(7):2239-2252. doi:10.1016/j.ymthe.2021.03.015.
5. Chi NF, Chiou HY, Chou SY, et al. Hyperglycemia-related FAS gene and hsa-let-7b-5p as markers of poor outcomes for ischaemic stroke. Eur J Neurol. 2020;27(8):1647-1655. doi:10.1111/ene.14288.
6. Munshi SU, Panda H, Holla P, Rewari BB, Jameel S. MicroRNA-150 is a potential biomarker of HIV/AIDS disease progression and therapy. PLoS One. 2014;9(5):e95920. Published 2014 May 14. doi:10.1371/journal.pone.0095920.
7. Goretti E, Rolland-Turner M, Léonard F, Zhang L, Wagner DR, Devaux Y. MicroRNA-16 affects key functions of human endothelial progenitor cells. J Leukoc Biol. 2013;93(5):645-655. doi:10.1189/jlb.1012511.

8. Rizzacasa B, Morini E, Mango R, et al. MiR-423 is differentially expressed in patients with stable and unstable coronary artery disease: A pilot study. PLoS One. 2019;14(5):e0216363. Published 2019 May 6. doi:10.1371/journal.pone.0216363.
9. Zhang Z, Wu S, Muhammad S, Ren Q, Sun C. miR-103/107 promote ER stress-mediated apoptosis via targeting the Wnt3a/β-catenin/ATF6 pathway in preadipocytes. J Lipid Res. 2018;59(5):843-853. doi:10.1194/jlr.M082602.
10. Shi FP, Wang XH, Zhang HX, et al. MiR-103 regulates the angiogenesis of ischemic stroke rats by targeting vascular endothelial growth factor (VEGF). Iran J Basic Med Sci. 2018;21(3):318-324. doi:10.22038/IJBMS.2018.27267.6657.
11. Natarelli L, Geißler C, Csaba G, et al. miR-103 promotes endothelial maladaptation by targeting lncWDR59. Nat Commun. 2018;9(1):2645. Published 2018 Jul 6. doi:10.1038/s41467-018-05065-z.
12. Huang L, Li L, Chen X, Zhang H, Shi Z. MiR-103a targeting Piezo1 is involved in acute myocardial infarction through regulating endothelium function. Cardiol J. 2016;23(5):556-562. doi:10.5603/CJ.a2016.0056
